# Canopy chlorophyll fluorescence applied to stress detection using an easy-to-build micro-lidar

**DOI:** 10.1007/s11120-019-00642-9

**Published:** 2019-05-25

**Authors:** Ismael Moya, Hildo Loayza, Maria Llanos López, Roberto Quiroz, Abderrahmane Ounis, Yves Goulas

**Affiliations:** 1 LMD/IPSL, CNRS, ENS, Ecole Polytechnique, Sorbonne Université, 91128 Palaiseau, France; 2 International Potato Center (CIP), P.O. Box 1558, Lima 12, Lima, Peru; 3 Innovation Department, AgriSat Iberia SL, 02007 Albacete, Spain; 4 Centro Agronómico Tropical de Investigación y Enseñanza (CATIE) Headquarters. Cartago, 30501 Turrialba, Costa Rica

**Keywords:** µ-lidar, LIF, SIF, Stress detection, LEDFLEX

## Abstract

LEDFLEX is a micro-lidar dedicated to the measurement of vegetation fluorescence. The light source consists of 4 blue Light-Emitting Diodes (LED) to illuminate part of the canopy in order to average the spatial variability of small crops. The fluorescence emitted in response to a 5-μs width pulse is separated from the ambient light through a synchronized detection. Both the reflectance and the fluorescence of the target are acquired simultaneously in exactly the same field of view, as well as the photosynthetic active radiation and air temperature. The footprint is about 1 m^2^ at a distance of 8 m. By increasing the number of LEDs longer ranges can be reached. The micro-lidar has been successfully applied under full sunlight conditions to establish the signature of water stress on pea (*Pisum Sativum*) canopy. Under well-watered conditions the diurnal cycle presents an M shape with a minimum (Fmin) at noon which is Fmin > Fo. After several days withholding watering, Fs decreases and Fmin < Fo. The same patterns were observed on mint (*Menta Spicata*) and sweet potatoes (*Ipomoea batatas*) canopies. Active fluorescence measurements with LEDFLEX produced robust fluorescence yield data as a result of the constancy of the excitation intensity and its geometry fixity. Passive methods based on Sun-Induced chlorophyll Fluorescence (SIF) that uses high-resolution spectrometers generate only flux data and are dependent on both the 3D structure of vegetation and variable irradiance conditions along the day. Parallel measurements with LEDFLEX should greatly improve the interpretation of SIF changes.

## Introduction

Photosynthesis is the process by which plants absorb H_2_O from the soil and CO_2_ from the atmosphere to generate sugars and release O_2_. Light collected by photosynthetic pigments (mainly chlorophylls and carotenoids) is the source of energy. To assess plant development and growth at medium scale (e.g., canopy to parcel), it is necessary to monitor the photosynthetic activity at a comparable scale. Chlorophyll absorption exhibits a strong transition in the near infrared reflectance spectrum. This spectral change has been used for a long time to determine the amount of vegetation. For instance, the normalized difference vegetation index (NDVI): NDVI = (NIR−R)/(NIR + R), which compares the vegetation reflectance in the near infrared (NIR) and red (R) parts of the spectrum is widely used in remote sensing. Sellers (1987) was the first to show a link between NDVI, absorbed, and incident photosynthetically active radiation (PAR) as

$${\text{NDVI}} \approx ({\text{absorbed PAR}}/{\text{incident PAR}}).$$

Using remotely sensed NDVI as a proxy for the fraction of absorbed PAR, gross primary production (GPP) can be estimated as GPP = α × NDVI × PAR, where α is a conversion efficiency empirically determined in the field for each vegetation type (see Moya and Flexas 2012 for a review). However, NDVI fails to detect dynamic variations of photosynthesis rates, like those occurring during the day or under certain stress conditions (Running and Nemani 1988).

Another proposed reflectance index is the photochemical reflectance index (PRI): PRI = (ρ531−ρ570)/(ρ531 + ρ570), where ρ531 and ρ570 are the vegetation reflectance at 531 and 570 nm. This index uses two spectral bands in the green and yellow parts of the reflectance spectrum to track reflectance changes near 531 nm associated with the de-epoxidation of the violaxanthin pigment into zeaxanthin that takes place under excess light conditions (Gamon et al. 1992). Evain et al. (2004) showed that PRI correlates better with non-photochemical quenching, related to dissipation of energy excess, than with photochemical quenching (e.g., photosynthesis activity), and is a good indicator for stomata closure upon water shortage. Indeed, PRI has been considered as a potential indicator of water stress (Peguero-Pina et al. 2008; Goerner et al. 2009; Suarez et al. 2008; Suarez et al. 2009) or light use efficiency (Cheng et al. 2006; Drolet et al. 2008) and it is also influenced by seasonal changes in pigment contents and canopy structure (Gamon et al. 2004). Besides these first employed reflectance signals, chlorophyll fluorescence is another parameter that increasingly interests scientists.

Chlorophyll fluorescence (ChlF) can be regarded as a small “leak” occurring during energy transfer in light-harvesting antennae. It is a natural emission between 650 and 800 nm (with two maxima in the red and far-red part of the spectrum) which emanates from the two photosynthetic systems. As fluorescence emission competes with photochemical conversion and thermal deactivation, in vivo chlorophyll fluorescence is variable and its variations mirror photochemical changes. Plant fluorescence is then subject to changes according to environmental constraints: light, temperature, water, and nutrient supply, among others. Although small (less than 1% of absorbed radiation) vegetation fluorescence is an attractive proxy for remote assessment of photosynthesis. Chlorophyll fluorescence can be measured using active or passive methods.

### Active methods

In active methods, a modulated and/or spectrally selected source of light excites the chlorophyll molecules that fluoresce between 650 and 800 nm. Most of active systems in remote sensing make use of pulsed light (lasers, laser diodes, LEDs) in the microsecond or even picosecond time range together with a synchronized detection necessary to measure it under day light conditions. As an example, the PAM (Pulse Amplitude Modulation) fluorimeter (Heinz Walz, Effeltrich, Germany), based on the pioneering work of Schreiber (Schreiber 1986), has been the basis of a huge development of fluorescence measurements at the leaf level. A large number of publications refer to this family of instruments. Measurements can be done on the dark-adapted state (Fo) or under ambient light (Fs). Changes in Fs with PAR are related to the photosynthetic performance (Baker 2008; Maxwell and Johnson 2000; Schreiber 1998). Moreover, using saturating pulses, the maximum fluorescence level in the dark (Fm) or under natural light (Fm’) can be measured, from which the photosystem II photochemical yield can be calculated according to Genty et al. (1989). Notwithstanding, due to limitations of the light source for saturating pulses, measurements can only be performed on a reduced target area (≈ 1 cm^2^). During the last two decades, many efforts have been made to be less invasive and to perform measurements at several meters of distance using, for instance, a laser diode at ≈ 635 nm which can be more easily focused than LEDs by virtue of its higher radiance (Flexas et al. 2000; Evain et al. 2004; López Gonzalez 2015). It was then possible to monitor Fs on a fixed leaf continuously during several days or weeks, with a time resolution of a few seconds, and to follow the onset and demise of stresses (Moya and Cerovic 2004). Nevertheless, the illuminated area was still of the same order than the original PAM fluorometer, and to obtain an information at plant or canopy level, a greater number of individual leaves had to be sampled.

Specific LIDARs (light detection and ranging) were developed for vegetation fluorosensing using green (532 nm) or UV (355 nm, 337 nm) lasers and even with dual lasers (355 and 532 nm) (Hoge et al. 1983; Günther et al. 1991; Anderson et al. 1994; Cecchi et al. 1994; Goulas et al. 1997; Chekalyuk and Gorbunov 1995; Cerovic et al. 1996; Rosema et al. 1998; Ounis et al. 2001). Laser-induced-fluorescence (LIF) measurements were severely limited by the high power required to saturate Fm over an area of ≈ 1 m^2^ or more when working on canopies. To overcome these limitations, the laser-induced fluorescence transient (LIFT) method proposed to use fast repetition rate (FRR) of sub-saturating light pulses to retrieve an extrapolated Fm value from the analysis of the observed induction kinetics (Ananyev et al. 2005). On the other hand, eye-safety restrictions are 10 times more restricting when using excitation wavelengths above 400 nm. Most of remote-sensing fluorescence applications under field conditions are based on the measurement of F_S_. Even if these variations are lower than those of Fm, it can be up to 100% of the stationary value (Cerovic et al. 1996). Although most of these works were intended to demonstrate the possibility to detect chlorophyll fluorescence at a distance, they were implemented in indoors or under protected conditions and without studying any particular stress. An interesting exception was the work of Rosema et al. (1998) which established a fluorescence signature for drought on poplar plants. In this particular experiment, the samples were placed inside a greenhouse and chlorophyll fluorescence was analyzed by means of a laser set-up (Laser Environmental Active Fluorosensor, LEAF-NL). The Nd:YAG laser of the LEAF-NL instrument provided a 10-mJ per 10 ns duration pulse at 532 nm forming a spot hitting the plants of ≈ 60 cm of diameter. Continuous measurements during several days allowed Rosema et al. to evidence a strong Fs quenching (Fs < Fo) at noon under drought conditions that partially reverses during the night.

### Passive methods

Recently, new passive methods for quantifying ChlF at canopy level attracted a large audience. They are based on the existence of dark bands in the solar spectrum—the so-called Fraunhofer lines—which are well superimposed with the chlorophyll fluorescence emission spectrum (Moya et al. 1998) and often measured using the Fraunhofer Line Discrimination Principle (FLD). In short, the FLD compares the depth of the line in the solar irradiance spectrum to the depth of the line in the radiance spectrum of plants. Plascyk and Gabriel (1975) were the first to develop an airborne instrument (FLD II) principally used to detect fluorescence of rhodamine dye in water. In a second step, the method was envisaged for measuring steady-state sun-induced fluorescence (SIF) from plants, using the Hα line at 656.28 nm (Plascyk 1975).

A new option for measuring SIF was later developed by Moya et al. (1998) using the oxygen absorption bands. Compared to solar absorption lines, oxygen absorption bands have the advantage of being relatively broad, deep, and well superimposed with the two characteristic peaks of the fluorescence emission spectrum at 685 and 740 nm (Moya and Cerovic 2004). Several works using narrow interferential filters allowed Louis et al. (2005) to measure fluorescence at distances up to 50 m. Moya et al. (2006) quantified the fluorescence emission of fields at both 687 and 760, from an airborne platform with a filter-based home-made instrument and emphasized the strong effect of the tridimensional structure of the vegetation on the F687/F760 fluorescence ratio. The clear decrease of the F687/F760 fluorescence ratio on going from a planophile field (e.g., sugar beet) to an erectophile one (e.g., wheat) was later analyzed on ground by Fournier et al. (2012) using a spectrograph-based instrument which confirmed these results.

Thanks to the recent availability of compact, high-resolution spectrometers, a great number of SIF measurements have been conducted using the atmospheric oxygen absorption bands. Different setups were used from leaf to canopy level (Meroni and Colombo 2006, Meroni et al. 2009; Rossini et al. 2010; Cheng et al. 2013; Daumard et al. 2010, 2012; Fournier et al. 2012; Damm et al. 2010; Cogliati et al. 2015) or from an airborne platform (Zarco-Tejada et al. 2009; Damm et al. 2015; Rascher et al. 2015). In most of the cases, passive instruments based on spectrometers used an optical fiber with a numerical aperture of 0.22 (acceptance angle of 25°) which means a target size of about 1 m at a distance of 4 meters. This makes the method well adapted to work at canopy level and obviously under full sunlight conditions. However, SIF may show variations linked to the angular distribution of incident light, depending on canopy architecture (Fournier et al. 2012; Goulas et al. 2017), impeding the interpretation of SIF in terms of fluorescence quenching and physiological acclimation of plants. On the other hand, although active ChlF measurements provide a direct assessment of fluorescence quenching, as stated above, there is no simple active fluorimeter that can acquire fluorescence on a target large enough for canopy studies.

The aim of this paper is to describe an easy-to-built instrument, named LEDFLEX, that measures ChlF at canopy level requiring only a few skills in mechanics and electronics that are usually available in most of plant-physiology laboratories, and to evaluate its potential in plant stress detection. Our work aimed to:

- Design, build, and test a prototype of an active fluorometer, capable of performing measurements at several meters from the target, with a spot size large enough to integrate its spatial heterogeneity; and
- Record continuously the stationary ChlF (Fs) under outdoor conditions and full solar illumination, in order to obtain a plant status fluorescence signature. The possibility to obtain the fluorescence signature of a moderate (reversible) water stress, before pre-visual signs appeared, is discussed.

## Materials and methods

### Description of the LEDFLEX micro-lidar

**Fig. 1:**
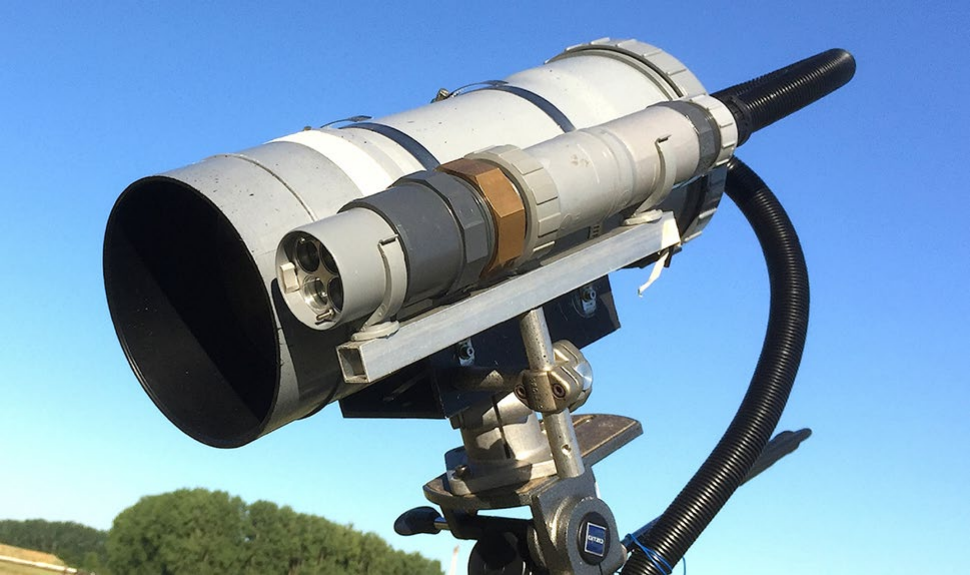
The LEDFLEX micro-lidar. The smaller tube contains the excitation source and the driver electronics. The larger tube contains the detection optics, the detector and its amplifier, the acquisition board, and the computer

We developed a micro-lidar (µLidar) system to monitor chlorophyll fluorescence from vegetation, an instrument composed of three main parts: (i) a light source, (ii) an optical telescope, and (iii) a detection system (Fig. 1). It was designed to work day and night under all weather conditions. All the detection parts were housed into a “drainage” pipe (polyvinyl chloride (PVC) tube of 160 mm diameter). The light source was contained within the 50 mm of diameter PVC pipe and connected to the detection pipe by a flexible 25 mm of diameter PVC pipe. The light source and telescope were fixed on two independent mounts and were mechanically adjustable, depending on the working distance.

### The excitation light source

Nowadays, there are many LEDs that fit our needs to induce ChlF provided that its wavelength emission is below 650 nm. We choose LED470L emitting at 470 ± 5 nm, with a full width at half-maximum (FWHM) of 22 nm (Thorlabs, Maisons-Laffitte, France) for the following reasons:

- Spectral purity (no emission in the red or infrared parts of the spectrum).
- Maximum continuous optical power 170 mW (at 350 mA).
- High-peak current > 3A at a frequency of 100 Hz for a pulse duration of 5 µs. The optical peak power should be > 1 W.
- Low power supply voltage (3.8 V).

Pulsed light is a pre-requisite to disentangle the weak fluorescence emission from the continuous daylight background. We chose to detect pulses of ≈ 5 µs of duration as it fitted well the time constant of our detection system (see below). As a result we pushed the current up to 3A without damage. We estimated the rise time of natural daylight variations (changes in illumination due to clouds) at a few seconds. Using pulses repetition rate of about 100 Hz and an averaging of the elementary measurements to a final frequency of ≈ 0.5 Hz to improve the signal-to-noise ratio, we were able to follow these daylight variations. Four LED470L LEDs were mounted in series in our instrument to obtain enough signal to measure at a distance up to ≈ 10 m, depending on the target (see below). In our experiments, the illuminated area was about 50 cm  × 50 cm at a distance of 4 m. Even in the dark, the blue light of LEDFLEX was found to be not disturbing at this distance—i.e., it did not drive any change in fluorescence and photosynthesis.

**Fig. 2:**
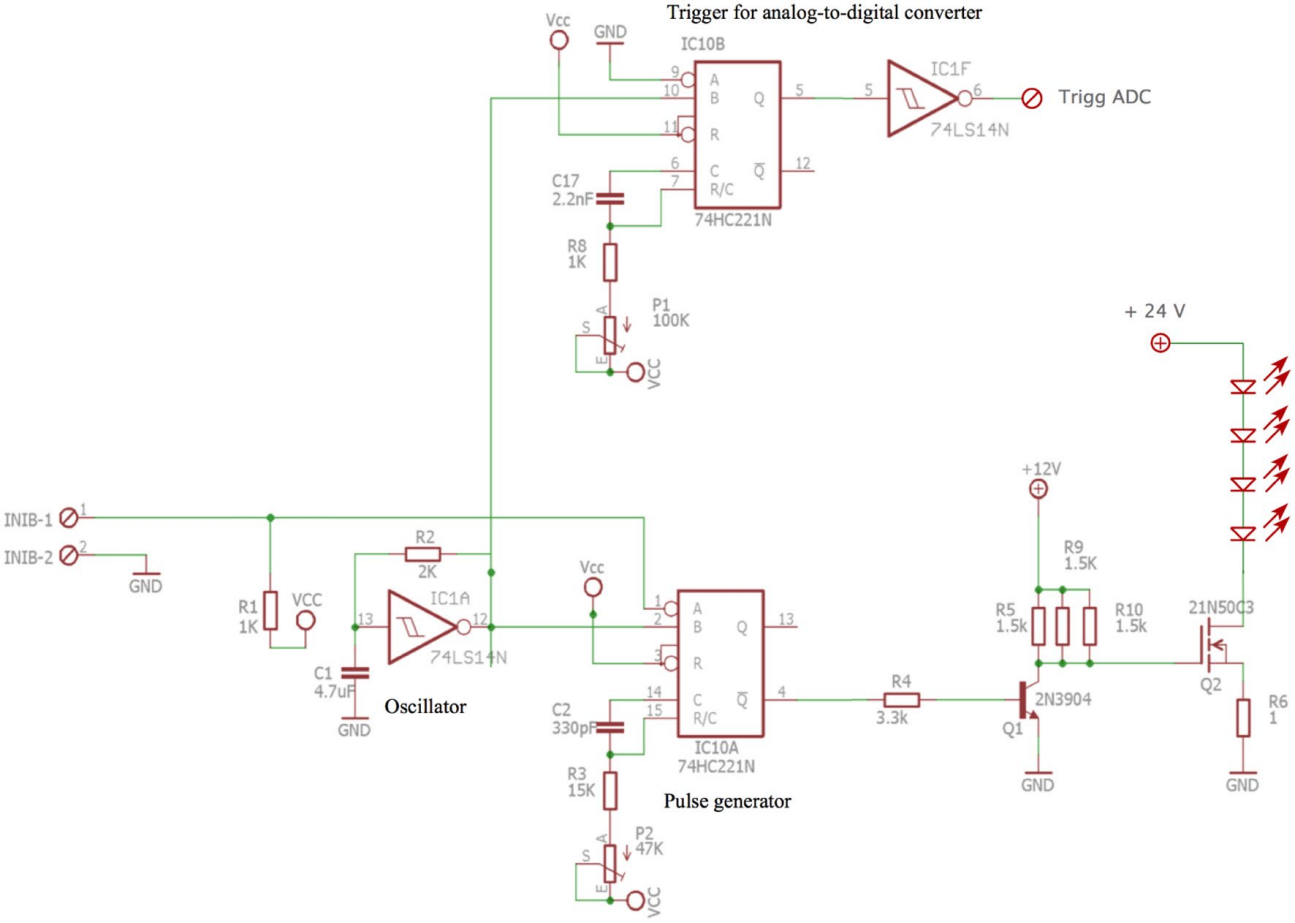
Scheme of the light source electronics. A frequency of ≈ 100 Hz is generated by the oscillator that feeds two monostables: the first one triggers the LED source and the second the acquisition board

The scheme for the electronic circuitry that drives light source is given in Fig. 2. The time base frequency was produced by a Schmitt-trigger inverter (IC1A; 1/6 74LS14 N, Texas Instruments, Dallas USA) inserted in a feedback loop including a RC (Resistor Capacity, with R = 2 kΩ and C = 4.7 µF, RC = 9.4 ms) cell to generate a square wave of approximately 100 Hz. The Schmitt-trigger output was fed to a first monostable (IC10A; 1/2 74HC221 N, Mouser electronics, USA) that defines an adjustable pulse duration of about ≈ 5 µs. The output of the first monostable was amplified by a transistor 2N3904 (Farnell, France) to generate a signal of ≈ 12 V, which in turn fed a 21N50C3 MOSFET (Infineon, Germany) that drove the current for powering the diodes. Four identical LEDs were mounted in series and required 24 V. To synchronize the LED emission and the detection, we generated a TTL signal that triggered an analog to digital conversion card (USB-6210, National Instruments, USA). This was achieved by a second monostable (IC10B, 1/2 74HC221 N) synchronous with the first one followed by a second Schmitt-trigger inverter (IC1F; 1/6 74LS14 N). For practical reasons, the whole instrument was powered by a unique source of voltage (12 V). The total power consummation of the whole instrument was < 1.3 A/12 V which can be easily powered by a solar panel. We generated 24 V using a DC–DC converter (12 V-24 V XP-POWER, USA) (not shown). A second DC–DC converter generated ± 5 V and ± 15 V using a DC/DC 12/5, ± 15 V (XP-POWER, USA) whose voltages were required to generate TTL signals and to power the OP27/37 amplifiers of the detection system. By adjusting the voltage of the MOSFET, we determined the current (3 A for each diode).

### The Optics

The fluorescence of the target was collected by a 152-mm diameter Fresnel lens (Edmund Optics, France) with a focal length of 76 mm and focused on a PIN photodiode (Hamamatsu S3590-01) after passing through a high-pass filter (Schott RG665, Edmund Optics, UK). In addition a low-pass filter (λ < 800 nm, Edmund Optics, UK) reduced the spectral range to the useful zone (650–800 nm) where chlorophyll fluorescence was emitted.

**Fig. 3:**
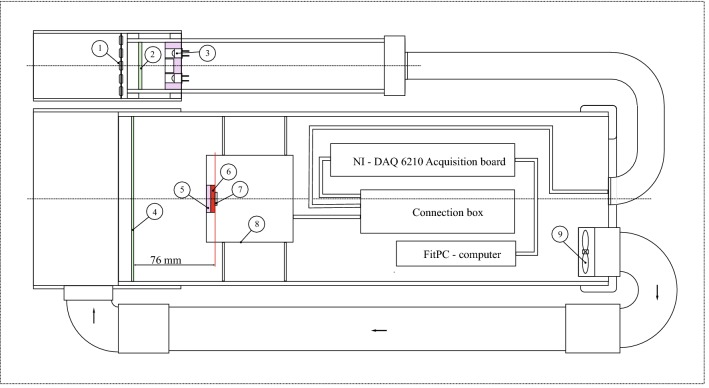
Schematic view of LEDFLEX. **1** Heating resistors, **2** Glass window, **3** Excitation LEDs, **4** Fresnel lens, **5** Low-pass filter (λ < 800 nm), **6** High-pass filter (λ > 660 nm). **7** 10 mm x10 mm Photodiode, **8** Detector housing, **9** Fan

To limit water condensation on the optical parts often occurring in the early morning, a heating system was added. Hot air produced by the computer and electronic components was extracted from the head by a small fan and directed by a PVC pipe to the outside face of the Fresnel lens (Fig. 3). This cooling of the electronics worked permanently and warmed the Fresnel lens on the outside. It was also necessary to warm the window in front of the LEDs. For this, we used a set of 5 11 Ω (0.5 W) resistors mounted in series and forming a ring through which the LEDs’ light crossed. The ring was placed outside, against the window protecting the LEDs in the sunshade. Powered by 12 V, they maintained the window at +10 °C above ambient temperature.

### The fluorescence detection system

**Fig. 4:**
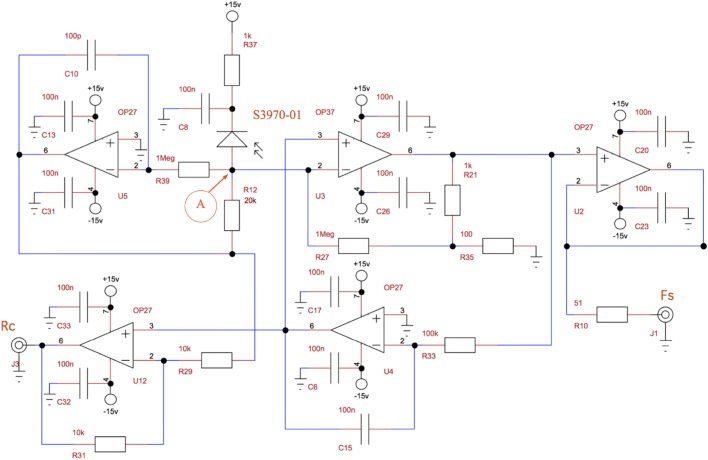
Electronics scheme for the detector. Basically two amplifiers (OP37 and OP27) are mounted as a low-pass filter in a feedback loop and generated both a pulsed output (Fs) and a near continuous signal (Rc) that accounts for ambient light (see text for details)

The fluorescence detection was designed to detect the fluorescence pulses generated by the LEDs’ excitation and at the same time be insensitive to ambient light. The light sensor was a PIN photodiode (S3590-01 Hamamatsu, France) of 10 × 10 mm that detected light in the range 300-1100 nm with a peak at 920 nm. It was reverse biased at 15 V. To detect the pulsed fluorescence, a circuit originally developed in the lab by M. Bergher (Fig. 4) used basically two amplifiers: a fast one (OP37, n° 1) and a slower one (OP27, n° 2 Analog Device, France) mounted as low-pass filter (cut-off frequency 1.6 kHz) in a feedback loop. As a result, the output of the slower amplifier corresponded to the averaged signal while the faster one only output the fluorescence pulses. A third OP27, (n° 3) was mounted as a low-pass filter (cut-off frequency 1.6 kHz) and maintained the potential of the photodiode cathode (point A, Fig. 4) near zero. Thanks to a fourth OP27, (n° 4), the amplitude of the low-frequency (f < 1.6 kHz) signal was formed by the algebraic difference between the outputs of the n° 2 and n° 3 amplifiers (low-pass filters) whose responses were in phase opposition. The low-frequency signal detected by the photodiode mainly consisted of the backscattered sunlight coming from the exact target place, seen in the spectral bandwidth of the fluorescence signal and the viewing direction of the sensor. Finally, a fifth OP27, (n° 5) was mounted as a follower to isolate the pulsed output from perturbations produced by the measuring devices. This detection system, used in our lab for more than 20 years, was the heart of our fluorimeter as it allowed to measure under full sunlight conditions.

A USB M Series 6210 multifunction DAQ board (National Instruments, USA) was used to collect and digitize fluorescence signals and other environmental parameters. A small computer FITLET-IA10-BB (D2 M, France) powered with 12 V and placed inside the main drainage pipe controlled the acquisition card and the data transfer between the card and the computer memory (see Fig. 3). The computer was programmed using HT BASIC 10 (TransEra Corporation, Orem, Utah, USA) for historical reasons but several other alternatives like C or Labview (National Instruments) exist. The computer was controlled over the internet to perform unattended measurements.

The 6210 board was configured with 8 differential channels. It had a single channel maximum rate of 250 kHz and an analog to digital conversion (ADC) resolution of 16 bits. One channel was devoted to the pulsed fluorescence measurement (OP27 n° 5, Fs). In order to be locked with the LED pulses, the output of the Schmitt-trigger IC1F (Trigg ADC, Fig. 2) was used to trigger the 6210 board to measure Fs at the peak value of the signal pulse. Here, a correct delay had to be adjusted. We used an oscilloscope to visualize both the TTL signal Trigg ADC and the Fs pulses. Then the instruction for measuring Fs at the peak was defined using steps of 50 ns. As the width of the Fs pulse was about several µs, this adjustment was not delicate. The other channels were operated in internal triggering mode and included measurements of the OP27 N° 4 output (Rc, Fig. 4), the Photosynthetic Active Radiation (PAR), detector temperature (Tdiode), LEDs temperature (Tled), and ambient temperature (Tair). Temperatures were measured using a thermistor (RS 151–237 Radiospares, France) of 10 kΩ at 25 °C fed by a 100 µA current source (REF 200, Texas Instruments USA). These environmental parameters were acquired each time an averaged Fs was stored (i.e., 2.27 s).

### Signal-to-noise ratio and working distance

**Fig. 5:**
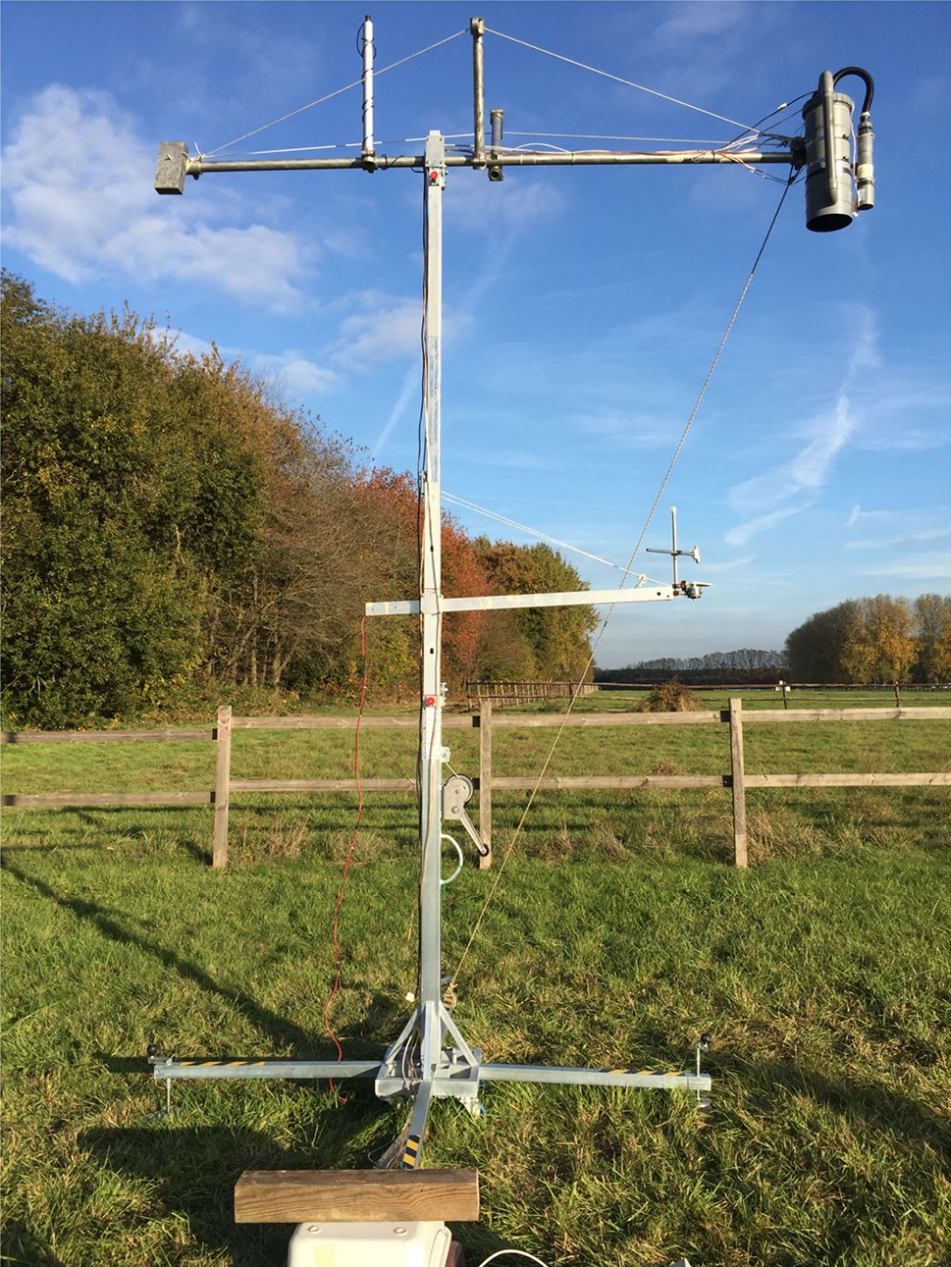
LEDFLEX in operation above grassland. The PAR sensor is fixed on a vertical rod above the boom. The boom is rigidified with ropes to avoid any movements due to wind

The moderate output power of the light source implied that the target could not be too far, as the detected flux decreased proportionally to the inverse of the square of the distance. We made some tests at midday, under full sunlight conditions, pointing the LEDFLEX head to nadir above the pea canopy. The ground surface was fully covered by pea leaves and the mean leaf chlorophyll content was about 40 SPAD units. At 4 m above the canopy, the signal was 0.8 V. The measured standard deviation of the Fs signal on 70 points (3 min of measurement) was ± 0.006 V. The signal-to-noise ratio (SNR) was 133, which we considered as good. During the night, the signal decreased to 0.72 V but with a much lower noise of ± 0.002 V resulting in a SNR = 360. The noise reduction at night was a proof that most of the noise during day was due to photon noise induced by ambient light. Given the quadratic decrease of the signal with distance, we expected an Fs value of 0.2 V at 8 m with the same noise. So, the SNR would drop to 33 under full sunlight, which was assumed to be still acceptable. However, we did not make the test at this distance because of the mechanical limitations of our set-up in a vertical position. Figure 5 shows the LEDFLEX complete set-up in the field.

### Plant material and environment

Experiments were carried out on the SIRTA (Site Instrumental de Recherche par Télédétection Atmosphérique) (48.7ºN, 2.2ºE) during summer and autumn of 2015. In a first experiment, plant material was *Pisum sativum* “petit provençal” variety. Seeds were planted outdoors, with a distance between them of approximately 2 cm in a pot (Ø 54 cm × 16 cm deep) with universal substrate. Measurements started when a homogeneous canopy was obtained. Several days after the beginning of measurements, water stress was induced by withholding watering. To monitor the recovery from water stress, the plants were irrigated again.

Measurements were also done on mint (*Mentha spicata*) canopy planted in pots as well as on Velvet grass (*Holcus lanatus*), a natural meadow. They were grown in outdoor conditions and maintained in well-watered conditions.

#### Water content index

To estimate a “water content index” (WCI), small leaf discs were extracted with a cork borer and weighted immediately to obtain fresh weight. These discs were immerged in distilled water at 4 °C and weighted after 24 h in the freezer to obtain the weight at full turgor. WCI was calculated following the relation:

$${\text{WCI}} = \, 100*\left( {\text{Fresh weight}} \right) \, / \, \left( {\text{Turgid weight}} \right).$$

According to Smart and Bingham 1974 there is an almost linear relationship between WCI and the relative water content defined as (RWC) = (Fresh weight−dry weight)/(Turgid weight−dry weight).

#### Gas exchanges measurements

The gas exchange measurements were made using the leaf chamber fluorometer of LI-6400 IRGA (Li-Cor, Lincoln, NE, USA). This chamber gives the quantum yield of PSII from chlorophyll fluorescence data in the same area where gas exchange is measured. Three to five attached leaves were measured at mid-morning during the different water treatments periods. The measurement conditions were constants with 1300 µmol m^−2^s^−1^ of PAR and 390 µmol CO_2_/mol of air concentration. The necessary measurement time was determined by the stabilization of the chamber parameters ([CO_2_] and [H_2_O]). According to the leaf sample, this time was between 2-3 min for well-watered plants and up to more than 5 min for stressed plants. All the measurements were carried out on fully expanded leaves at mid-morning. An additional stomatal conductance measurement at leaf level was obtained with a leaf porometer (Decagon Devices Inc., Pullman USA) thus increasing the number of leaves measured during the water stress induction.

#### Chlorophyll content

The chlorophyll content was estimated in SPAD units with the SPAD-502 (Minolta) over the campaign as the average of 20-30 measurements on different leaves.

#### PAR measurements

Photosynthetic Active Radiation was measured with a quantum-meter (JYP 1000, SDEC, Reignac sur Indre, France) fixed above the instrument in order to avoid any shade induced by the surrounding (Fig. 5).

## Results

### Pea measurements

Measurements at canopy level require spatial integration to average the fluorescence signal over a great number of leaves. It involves also measurements at distance in order to be non-invasive. We choose to use plants in a pot, as described in the methods, for a better control of water content. To achieve this, the canopy of pea plants that were grown outdoors (full sunlight) was used to perform measurements. Plants were sowed on August 18^th^ and maintained until October 6th. LEDFLEX was fixed in a vertical position 2.5 m above the target. Plants were well watered until September 8th, when we stopped irrigation.

### Control measurements

**Fig. 6:**
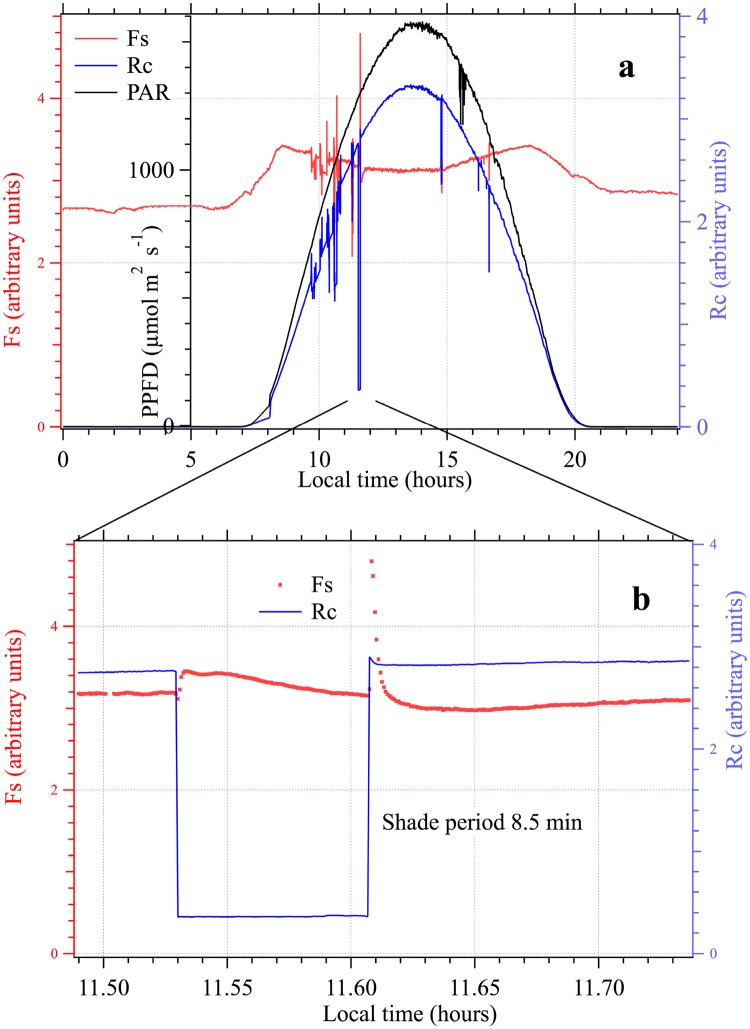
**a** Diurnal cycle of PAR, Rc (reflected light), and Fs (stationary fluorescence) above a pea canopy. **b** Detail of an artificial light transition. One may observe the small transient of Rc after the end of the shade period which is due to the fluorescence contribution

On September 10th the plants could still be considered as well-watered. Figure 6a shows, for this sunny day, a complete cycle of stationary fluorescence (Fs, red line) together with the reflected light (Rc, blue line) and measured Photosynthetic Active Radiation (PAR, black line). During the night, Fs stayed almost constant. This constant night level is denoted as Fo hereafter. After the night Fs increased with the first photons reaching the canopy until 8:30 a.m. Maximum of Fs was attained with a PAR of ≈ 250 µmol m^−2^s^−1^. Further PAR increases until solar noon (2:00 p.m.) led to a continuous decrease of Fs. Compared to the Fo value obtained during the night, Fs remained significantly higher (Fs solar noon > Fo). During the afternoon, Fs increased again to attain a second maximum (6:00 p.m.) equal to the one obtained in the morning. Notwithstanding, the PAR was still about 600 µmol m^−2^ s^−1^. Observed Fs signal variations between 9:30 and 11:00 a.m. were a consequence of the gas exchange measurements that caused disturbances in the measuring area, due to the limited size of the target.

To illustrate the LEDFLEX capacity to monitor rapid changes under changing illumination conditions, we produced an artificial shade of the target around 11:30 a.m., which reduced the incoming radiation to ≈ 13% of full sunlight (Fig. 6b). During the shade period lasting 8.5 min, we observed an increase of Fs followed by a decrease to a value close to that reached at full sunlight. When the shade was removed, a large increase of Fs was observed followed by a decrease to a level lower than under the shade. The increase of Fs depended, among other things, on the temporal resolution of the instrument (≈ 2.3 s) and on the speed at which the panel producing the shade was removed. After a few minutes, the effect of the shade completely disappeared.

### Stress measurements

Water stress was induced by withholding watering. The water content index (WCI) was estimated as described in the methods. Leaf discs were taken periodically in the early morning to avoid differences in water content due to water loss during the day. Environmental heterogeneity was present during the experiment, with sunny and cloudy days, as well as sunny and cloudy intervals within the same day. During rainy days the pots were covered.

**Fig. 7:**
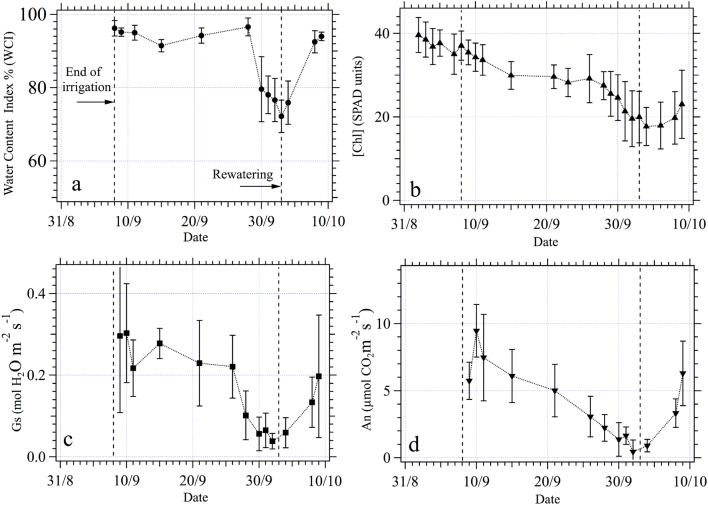
Pea canopy during water stress treatment. **a** Water content index. **b** Chlorophyll concentration. **c** Stomatal conductance (Decagon data). **d** Assimilation (LI-6400)

Figure 7 presents the evolution of several important physiological parameters recorded during the campaign that includes water content index (WCI), chlorophyll concentration [Chl], stomatal conductance (G_s_), and CO_2_ assimilation (An). WCI (Fig. 7a) started with a value of ≈ 90% that changed very little until September 20th, then decreased to ≈ 72% upon water stress and increased suddenly to ≈ 85% upon re-watering. Measurements started September 7th with a chlorophyll content of ≈ 37 SPAD units that decreased continuously until a minimum of 19 SPAD units when water stress was maximum and recovered partially (≈ 21 SPAD units) after re-watering (Fig. 7b). Stomatal conductance dropped from ≈ 0.3 at the beginning of the experiment (control) to 0.05 mol H_2_O m^−2^s^−1^ under stress. Re-watering restored stomatal conductance to ≈ 0.2 mol H_2_O m^−2^s^−1^ (Fig. 7c). The stress period was prolonged until assimilation fell from ≈ 8 µmol CO_2_ m^−2^ s^−1^ to almost zero (Fig. 7d). Then, re-watering was initiated and assimilation recovered up to ≈ 6 µmol CO_2_ m^−2^s^−1^.

**Fig. 8:**
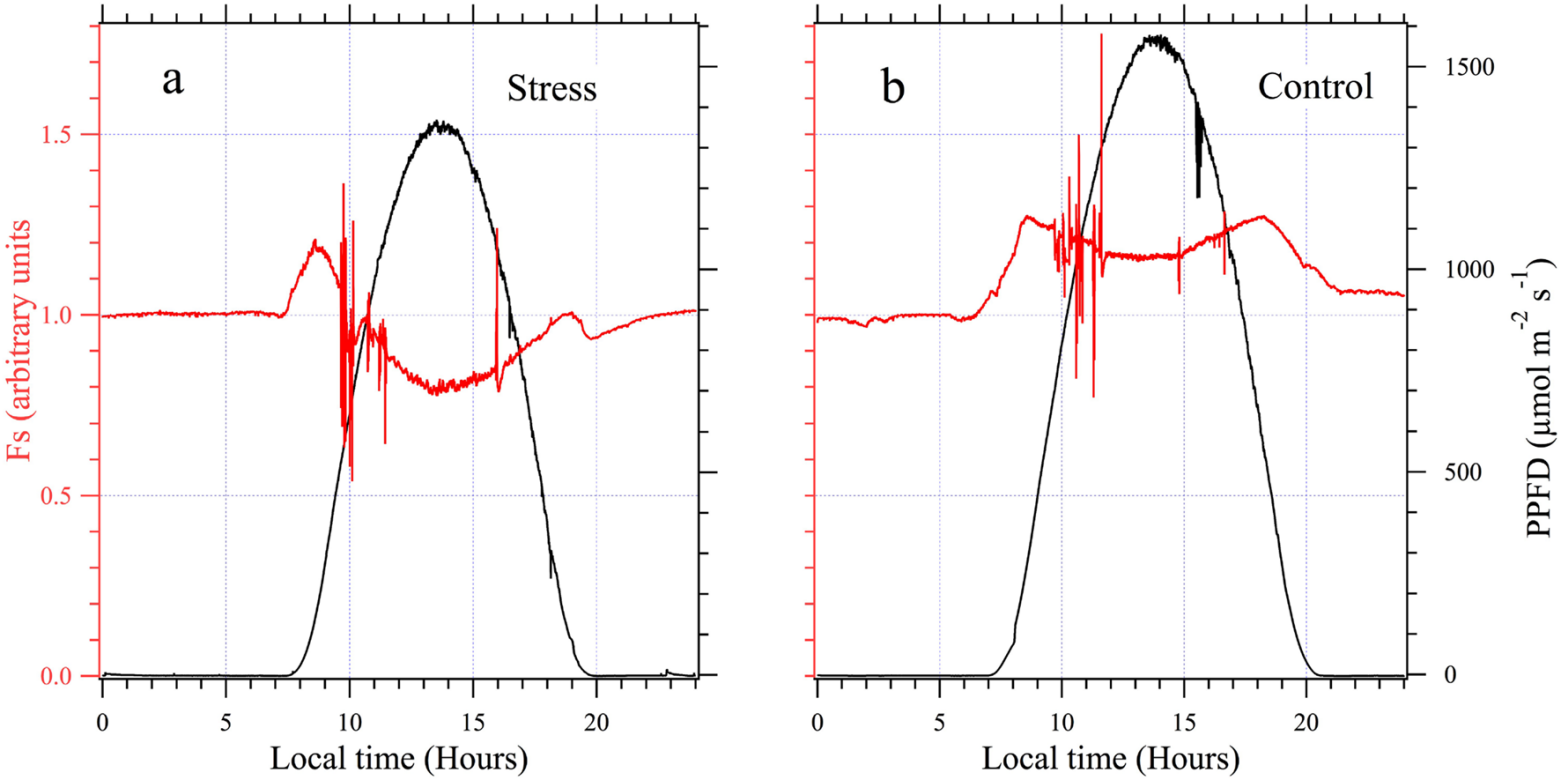
Diurnal cycles of chlorophyll fluorescence. **a** After several days withholding watering. Observe the decrease of Fs at noon that becomes lower than during the night (Fo). **b** The same well-watered pea canopy for comparison

To illustrate the changes that occurred in the Fs signal under stress conditions, a sunny day (October 1st) was chosen (Fig. 8a) to compare with the control cycle of September 10th (Fig. 8b). Fs measurement was partially perturbed between 9:30 and 11:00 due to interference with gas exchange measurements as evoked above. The Fs signal stayed constant during night (Fo) and increased early in the morning, like in the control case, until 8:30 a.m. However, as PAR exceeded 120 µmol m^−2^ s^−1^, Fs decreased continuously well below Fo, to reach a minimum of 78% of Fo at solar noon. During the afternoon, Fs increased but stayed below Fo. So we observed a large difference between control (Fig. 8b) and stressed plants (Fig. 8a) that can be characterized by the ratio between the peak of Fs in the morning ($F_{s}^{1}$) and the minimum at solar noon ($F_{s}^{2}$). This ratio changed from 1.1 (control) to 1.49 (stress).

### Mint measurements

**Fig. 9:**
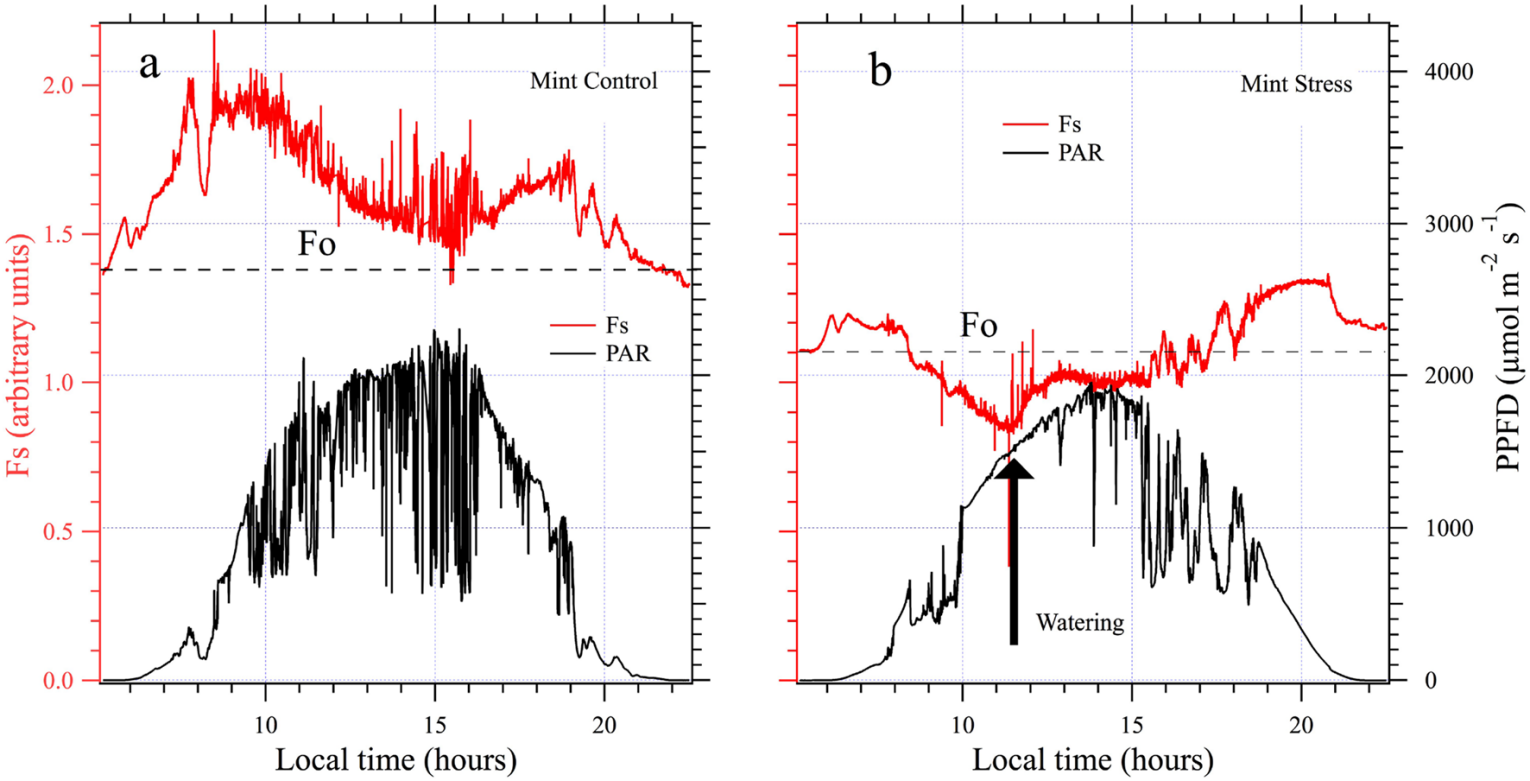
**a** Control mint cover a windy day. **b** Stressed mint cover. The rapid Fs decay has been reverted in minutes after watering

In order to test LEDFLEX with a different crop, we conducted, during two weeks, a campaign on a mint cover formed by 20 pots put together to form a target. At variance with the pea cover, the chlorophyll content stayed constant at 40 ± 4 SPAD units during the whole campaign (not shown). Assimilation and conductance measurements done with the Li-cor 6400 revealed values of An = 19 ± 4 µmol CO_2_ m^−2^ s^−1^ and Gs = 0.23 ± 12 mol H_2_O m^−2^ s^−1^, respectively, for well-watered plants (Fig. 9a). Although illumination conditions were not as constant as for peas (alternating clouds and sun), the diurnal cycle of Fs was similar to that of pea crop, M-shaped, but with a minimum at noon, well over the predawn level.

After 2 days withholding watering (Fig. 9b) we observed, in the morning, a small increase of Fs with low light rapidly followed by a strong decrease, well below Fo, when the illumination reached 500 µmol m^−2^ s^−1^. At 11:30, fearing that the strong sun damaged irreversibly the plants, we decided to irrigate. After a few minutes Fs increased, then became higher than Fo and continued to increase despite high light conditions (PAR > 1800 µmol m^−2^ s^−1^). The responsivity of the Fs signal also increased when light changed, for example, between 15:00 and 19:00 compared to morning conditions.

These observations made on peas and mint canopies were similar to those on sweet potatoes (*Ipomea batata*) made in Lima (Peru) when testing LEDFLEX (not shown).

### Grassland measurements

LEDFLEX was installed pointing nadir at 3.2 m above a natural grassland. Experiments took place at the beginning of November 2016 with rain every 4 or 5 days and rare sunny days. The target was principally composed of velvet grass (*Holcus lanatus*, *Holcus mollis*) of about 4–5 cm height. The target contained also a large amount of dried material and debris produced by previous grass mowing. The average chlorophyll content was about 30 SPAD units.

**Fig. 10:**
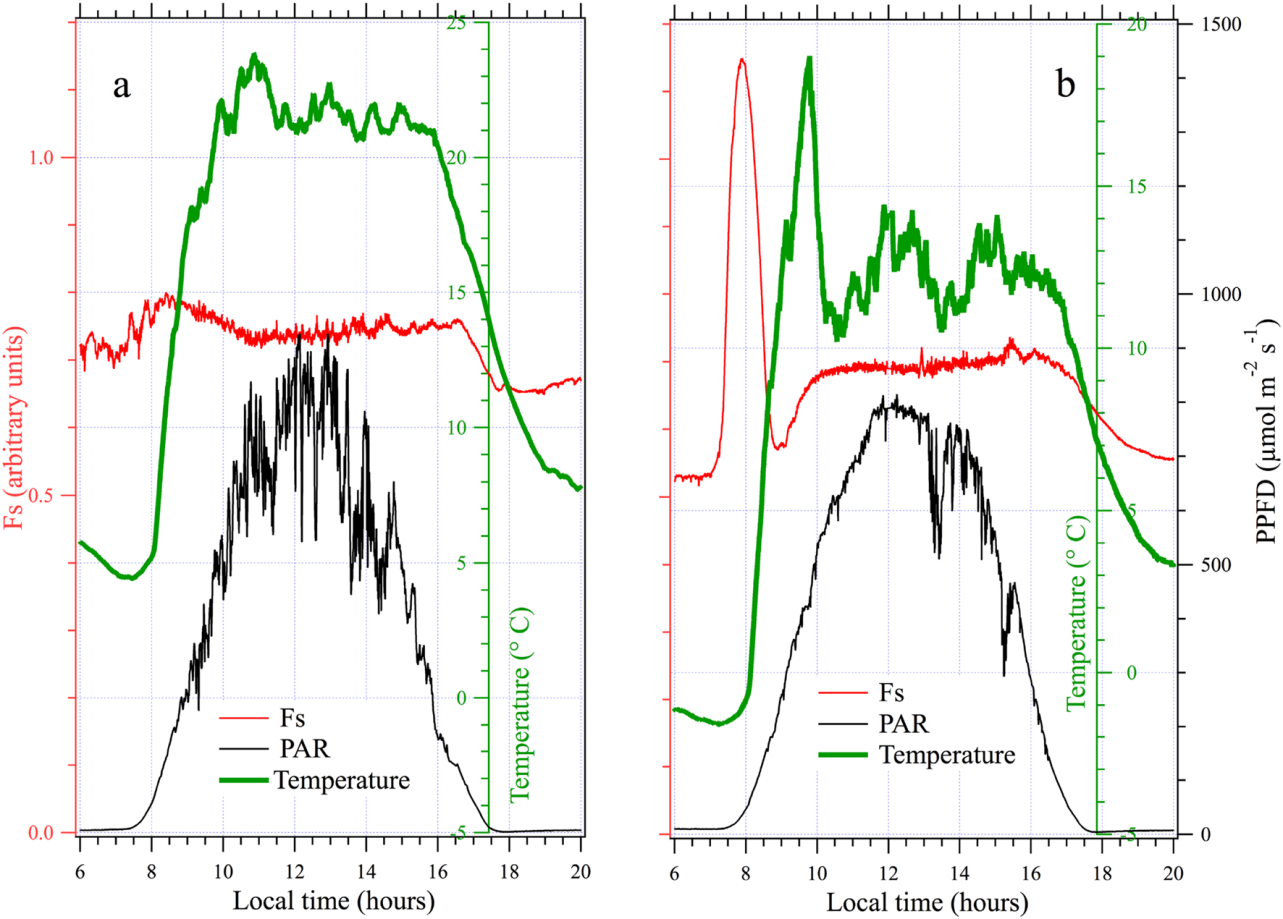
**a** Diurnal cycle of a natural grassland. **b** Diurnal cycle during a cold morning with negative temperatures at dawn

Figure 10a presents a rather sunny day (November 1^st^). One can appreciate the low extent of Fs variations and a general pattern rather similar to what was observed for potted pea plants. In the following days, the temperature tended to decrease with a concomitant decrease in Fo. Figure 10b shows what happened when temperature became negative during night (November 3^rd^). We observed a huge increase in Fs (more than twice!) in the early morning which reversed when the temperature became positive. This peculiar increment lasted about one hour, followed by a diurnal cycle similar to Fig. 10b. This behavior—a prominent peak occurring in the morning—has been observed each time the temperature was negative at dawn.

## Discussion

Our methodology consists of measuring continuously several parameters including stationary fluorescence Fs, reflectance of the target Rc, PAR, and temperature. These data are acquired from a fixed place and maintained unchanging the targeted part of the vegetation. Also Fo is measured daily. We focus on the eventual changes of the diel cycle that could be a stress signature. The experiments may last several weeks. Although non-commercial, the instrument can be built for a cost of less than 2 000 $. The technique can be applied to the study of other stresses, including nitrogen deficiency, albeit we did not have the opportunity to do such work. Notwithstanding, other excitation wavelengths seems more adapted to study nitrogen deficiency. For example, excitation in the UV generates a blue fluorescence emission proceeding from phenolic compounds together with the chlorophyll fluorescence emission (see Cerovic et al. (1999) and Tremblay et al. (2012) for a revue).

### Water stress effect on Fs

Other authors have measured the effect of water stress on fluorescence. Cerovic et al. (1996) using a modified PAM 101 (Walz, Effeltrich, Germany) monitored Fs at a distance up to 1 m on an attached leaf. The authors monitored several species submitted to drought including maize, sugar beet, and kalanchoë. On maize, after six days withholding watering Fs decreased at noon to a value lower than Fo. Although in this experiment the light intensity was limited to less than 350 µmol m^−2^ s^−1^ for technical reasons, these results are in line with the data presented here.

Flexas et al. (2000) studied also the effect of water stress on an attached leaf of a vine plant, during a campaign of 17 days. The authors developed a new fluorometer based on a laser diode for measuring at distance both Fs and F’m through the window of a Li-cor 6400 gas analyzer. They also evidenced the M shape of the diurnal cycle of Fs with a minimum at solar noon. Under stress conditions, the evening branch was much lower than the morning one and the minimum ($F_{s}^{2}$) was clearly lower than Fo, in agreement with what is shown in Fig. 8 of the present paper. Other works including Ounis et al. (2001) and Evain et al. (2004) reported similar results. However, in the above-mentioned papers only a single point of the leaf was analyzed.

Rosema et al. (1998) used a target formed by poplar trees grown in pots in a growth cabinet with glass walls inside a greenhouse. A Nd-Yag laser providing pulses of 10 mJ of 10 ns length at 532 nm was used for excitation. The laser illuminated an area of 60 cm of diameter at 12 m. During a water stress experiment lasting 5 days, the diurnal cycle showed a dip at noon that developed and became lower than Fo when drought progressed. Indeed, inside a greenhouse with low radiation (< 400 µmol m^−2^ s^−1^), the water stress signature was evidenced at canopy level. Although, unnoticed to the authors, a small peak on Fs can be observed at dawn in Fig. 7 of their publication, as it is evidenced in the present work.

Bright light conditions prevailed in an outdoors vineyard work presented by López Gonzalez (2015). They used a laser-diode µLidar, developed at LMD (Laboratoire de Météorologie Dynamique, Paris), which was able to measure Fs from a distance of a few meters, over a plant section containing several leaves. Field work was conducted during the summer, for 45 days, at Barrax, in the South of Spain. Fs was continuously measured from well-watered conditions (Gs = 0.18 mol H_2_O m^−2^ s^−1^) to stress conditions (Gs = 0.05 mol H_2_O m^−2^ s^−1^). During this long period of good weather, neither the chlorophyll content nor the reflectance were modified. The authors observed a progressive decrease of Fs at noon, which dropped below Fo at the end of the treatment. Importantly, 12 h after re-watering, a diurnal cycle similar to control plants was obtained. These results were very similar to those obtained on mint plants or peas and presented in this work, although they were obtained with different plant species.

### Effect of negative temperatures at dawn on Fs

LEDFLEX uses the shelf supplies and is robust, sensible, and efficient. It can measure fluorescence outdoors continuously, during prolonged periods of time, under full sunlight conditions. It is worth noting that the installed warming system allowed us to measure under negative temperatures (°C) without water condensation on the front-end windows.

Under normal conditions (positive temperatures and well-watered plants) we observed a very small peak (≈ 5% of Fo) that often occurs at dawn, when the first photons reached the system. We interpreted this small increase as a transitory reduction of Q_A_ occurring when light hits the target. It disappeared after complete activation of photosynthesis. This small peak at dawn has also been observed by Flexas et al. (2000) and by López Gonzalez (2015). Under “normal conditions,” Fs variations were between 5 to 20% of Fo. When temperature was low at dawn, enzymatic reactions were slower and a larger reduction of Q_A_ occurred as soon as light increased. This phenomenon ended when temperature increased; in turn, following further increase of daylight after 8:00 a.m., allowing the reoxidation of Q_A_. We did not find any other reference in literature describing this phenomenon.

The effect of cold and light stress on photosynthesis parameters was studied using the LIFT approach (Pieruschka et al. 2010). An impairment of the photosynthetic efficiency was observed on some species under cold stress including *L. esculentum and C. annuum*. However, in other species like grass the authors did not report any change on the fluorescence under cold treatment. In particular they did not observe our dawn occurring peak. Compared to LEDFLEX, LIFT used high intensity light pulses with high duty cycle periods to saturate photosynthetic activity within PSII reaction centers. To avoid accumulation of possible harmful excess light, sampling frequency is necessarily limited, so rapidly occurring events can be missed with the LIFT approach. This is not the case with LEDFLEX which continuously samples stationary fluorescence level at high rate. The sampled area is also larger with LEDFLEX (around 1 m^2^ at 8 m distance) compared to LIFT (around 100 cm^2^).

## Conclusions

We presented in this work a non-commercial instrument dedicated to continuous measurement of chlorophyll fluorescence of vegetation under natural conditions. All the electronics and optics were encapsulated within a PVC drainage pipe of 160 mm of diameter and 550 mm length connected to a solar panel by a single 12-V power line. Data were collected using a wireless connection. The measuring range was more than 8 m, depending on the target, but it can be increased easily using a greater number of diodes.

A simple signature of water stress emerged, based on a strong increase (35%) of Fs(9 h) − Fs(noon) on stressed plants compared to the control, associated with a Fs(noon) below Fo. These facts have been established for several crops. Thanks to a simple warming system, it was possible to work with negative ambient temperatures that revealed new fluorescence features like a conspicuous peak at dawn.

The device has been duplicated and currently applied for the study of potatoes and sweet potatoes, in parallel with a spectrometer-based passive instrument in Peru.

## Abbreviations

NDVI: Normalized difference vegetation index
NIR: Near infrared
R: Red
PAR: Photosynthetic active radiation
GPP: Gross primary production
PRI: Photosynthetic reflectance index
ρ531: Reflectance at 531 nm
ρ570: Reflectance at 570 nm
ChlF: Chlorophyll fluorescence
LED: Light-emitting diode
PAM: Pulse amplitude modulation
Fo: Minimum dark-adapted fluorescence yield
Fs: Stationary fluorescence yield
Fmin: Minimum of stationary fluorescence at around solar noon
Fm: Maximum dark-adapted fluorescence yield
Fm’: Maximum steady-state fluorescence yield
LIDAR: Laser-induced detection and ranging
UV: Ultra-violet
LIF: Laser or led-induced fluorescence
SIF: Sun-induced fluorescence
PVC: Poly vinyl chloride
TTL: Transistor–transistor logic
DAQ: Data acquisition
ADC: Analog to digital conversion
Rc: Reflected light
Tdiode: Detector temperature
Tled: Light-emitting diodes temperature
Tair: Air temperature
SNR: Signal-to-noise ratio
PPFD: Photosynthetic photon flux density
FWHM: Full width at half-maximum
